# A Novel Nano Magnetic Beads Dot ELISA Immunoassay and Its Application on the Detection of *Giardia lamblia* Coproantigen

**Published:** 2018

**Authors:** Nagwa SHABAN ALY, Ibrahim BAYOUMI, Rabab SELEM, Manal KARDOUSH, Gehan RASHED, Ahlam MOHARAM

**Affiliations:** 1. Dept. of Parasitology, Faculty of Medicine, Benha University, Benha, Egypt; 2. Dept. of Immunology and Evaluation of Drug, Theodor Bilharz Research Institute, Ciza, Egypt

**Keywords:** *Giardia lamblia*, Dot ELISA, Nano technology

## Abstract

**Background:**

The traditional basis of diagnosis is identification of *Giardia lamblia* trophozoites or cysts in the stool of infected patients. Recently the advent of more objective techniques like antigen detection methods has led to an increase in their use versus those that rely on subjective microscopic examination of fecal specimens for *Giardia* cysts may facilitate diagnosis of *G. lamblia* in stool specimens.

**Methods:**

This cross-sectional study was carried out from Oct 2015 to Feb 2016 on patients admitted to Benha University Hospitals (Benha, Egypt) and outpatients of Theodor Bilharz Research Institute (TBRI) (Giza, Egypt). Purified *G. lamblia* cysts antigen was prepared by two-phase sucrose gradient technique. Polyclonal antibody against purified *G. lamblia* cysts antigen was prepared and labeled with horseradish peroxidase and Nano Magnetic Beads (NMB) to be used as detecting antibody. A total of 72 stool samples, 32 samples positive for giardiasis, 20 samples positive for other parasitic infections in addition to 20 negative samples were examined using dot ELISA and NMB dot-ELISA.

**Results:**

The sensitivity of the traditional dot-ELISA was 81.3 % and it increased by using the NMB-dot-ELISA to be 96.9% in stool samples. Specificity of both techniques was 97.5%.

**Conclusion:**

Diagnosis of *G. lamblia* by NMB-Dot-ELISA technique is sensitive, specific, rapid and easy to perform and interpret. In this study, using the nano-magnetic beads increased the sensitivity of the applied technique.

## Introduction

*G. lamblia* is one of the most common intestinal protozoan parasites, affecting ∼200 million people in Africa, Asia, and Latin America. More than 280 million people are at risk of acquiring this disease (1). Symptoms typically develop 9–15 d after exposure (2). They vary from none to severe diarrhea with poor absorption of nutrients, weakness, loss of appetite, stomach cramps, vomiting (uncommon), bloating, excessive gas, and burping. Giardiasis occurs after transmission of *G. lamblia* cysts through the ingestion of contaminated water and food. The infection may also be transmitted from domestic animal to man (3).

Microscopic examination for the presence of cysts in stool is the most straightforward and widely used diagnostic method to test for giardiasis infection. Many methods have been investigated for automating the detection of *Giardia* spp., including enzyme immunoassay, immunofluorescent assay, and counter-immunoelectrophoresis. An enzyme-linked immunosorbent assay (ELISA) detects secretory and excretory of the organism is now available. The test can be completed in less than 3 h and does not depend on simple equipment for interpretation (4).

Dot enzyme-linked immunosorbent assay (Dot-ELISA) is a versatile solid-phase immunoassay for antibody or antigen detection. It uses minute amounts of reagent dotted onto solid surfaces which avidly bind proteins. After incubation with antigen-specific antibody and enzyme-conjugated anti-antibody, adding of chromogenic substrate causes formation of a colored dot visually read (5, 6). The Dot-ELISA has been used extensively in the detection of parasitic diseases, including amebiasis, malaria, toxoplasmosis, babesiosis, cutaneous and visceral leishmaniasis, trypanosomiasis, schistosomiasis, toxocariasis, fascioliasis, cysticercosis, echinococcosis, trichinosis, and even ixodid tick infestation (7,8).

The use of metal nanoparticles has been expanded in biomedical research. They have been used for diagnostic and therapeutic purposes due to their unique characters of small size, high reactivity to the living cells and stability over high temperatures. They are available in different sizes (10–100 nm) and shapes (9). Most commonly studied metal nanoparticles include gold, silver, titanium oxide and iron nanoparticles (10). For detection of *G. lamblia* antigen in stool samples nano-gold beads ELISA was previously used (8).

This works aimed to develop novel nano diagnostic assays (nano-magnetic beads based-ELISA and nano-magnetic beads dot-ELISA assays) for detection of coproantigen in stool of human samples of patients infected with *G. lamblia* and compared them with the traditional sandwich ELISA. This study represents the first research uses NMB-dot ELISA in detection of *G. lamblia* antigen in stool samples of infected patients.

## Materials and Methods

### Sample collection

This cross-sectional study was carried out from Oct 2015 to Feb 2016 on patients admitted to Benha University Hospitals (Benha, Egypt) and outpatients of Theodor Bilharz Research Institute (TBRI) (Giza, Egypt). Written informed consents were obtained from all patients. Fecal samples from a total 72 patients were assigned into three groups based on microscopic stool examinations. Group I included patients positive to *G. lamblia* (n= 32) and Group II included patients with parasitic infections other than giardiasis (n= 20). Group III consisted of controls, healthy and free-intestinal parasites (n = 20). These samples had been screened using a direct smear and Merthiolate iodine formaldehyde concentration methods (MIF) (11).

Each fecal sample was processed by mixing the fecal material in a 1:5 proportion with PBS-formalin 5%. The samples were mixed by a vortex to form slurry then centrifuged at 3000 ×gr for 30 min at 25 °C. The supernatant was recovered and stored at 4 °C until used. On the day of using, supernatants were mixed by vortex and re-centrifuged at 3000 ×gr for 15 min before use (12).

### Preparation of G. lamblia antigen

Purification of *G. lamblia* cysts using two-phase sucrose gradient technique: The cysts from fecal samples were collected from patients diagnosed with giardiasis. Cysts were purified. The *Giardia* cysts were washed three times with phosphate-buffered saline (PBS), homogenized and centrifuged at 500 × *g* for 1 h at 4 °C. The protein content was measured using Bio-Rad Protein Assay (Bio-Rad, Hercules, USA) according to the manufacturer’s instructions and the antigen was stored at −20°C until assayed.

### Production and purification of polyclonal antibodies

#### 1. Immunization of rabbits for production of polyclonal antibodies

Blood samples were collected from healthy rabbits before injection and examined with ELISA for checking for *Giardia* antibodies and cross-reactivity with other parasites (13). Rabbit’s anti-*giardia* serum was obtained by immunizing New Zealand white rabbits (approximately 1.5 kg weight) with *Giardia* antigen. One mg of purified *Giardia* antigen was given to each rabbit in entire course of immunization. The rabbit received priming dose intramuscular injection (IM) at four sites, 1mg purified *Giardia* antigen mixed with equal vol. of complete Freund’s adjuvant (CFA), (Sigma). Three booster doses were given, each was 0.5 mg antigen emulsified in equal vol. of incomplete Freund’s adjuvant (IFA), (Sigma). The first boosting was two weeks after priming dose. The following boosting doses were given at weekly intervals (14). The rabbit was bled for collection of serum after three days from last dose. Rabbit serum which contains anti-*Giardia* polyclonal antibody (pAb) IgG was fractionated and kept at −20 °C. Test blood samples were withdrawn before the injection of each immunization dose to detect the titer of antibodies (Ab) produced by the color.

#### 2. Labeling of polyclonal antibody with Horseradish peroxidase (HRP) (Periodate method)

Five mg of horseradish peroxidase (HRP) (Sigma) was resuspended in 1.2 ml of dist. H2O. 0.3 ml of freshly prepared sodium periodate was added and incubated at room temperature for 20 min. The HRP solution was dialyzed against 1 mM sodium acetate buffer, pH 4 at 4 °C with several changes overnight polyclonal solution of 5 mg/ml in 0.02 M carbonate buffer, pH 9.6 was prepared. The HRP was removed from dialysis tubing and was added to 0.5 ml of antibody solution. The mixture was left to incubate at room temperature for 2 h. Hundred μl of sodium borohydride was added and the solution was incubated at 4 °C for 2 h. The HRP-antibody conjugate was dialyzed with several changes against 0.01 M PBS, pH 7.2 (15).

#### 3. Loading of Nano-Magnetic Beads (NMBs) to polyclonal antibody (pAb)

Nano-magnetic particles (usually <40 nm in size) are inorganic based particles having an iron oxide core coated by either inorganic material (silica, gold) or organic materials (phospholipids, fatty acids, peptides or other polymers). Beads are micro-sized polymer particles loaded with the nanomagnetic particles. Such beads can be functionalized with molecules that allow specific adsorption of proteins or other biomolecules with the ability of subsequent separation in a magnetic field gradient for diagnostic purposes.

NMBs were prepared and conjugated with Ab, 5mg of the prepared anti- *Giardia* IgG-PAb in phosphate-buffer, pH 7.5 were added to 10ml pH adjusted NMBs solution. The mixture was gently mixed for 3 h, and subsequently, 2 ml of 10% BSA solution was added to block the residual surface of the NMBs. The mixture was then incubated for 20 min at room temperature before being centrifuged at 13000 gr for 45 min at 4 °C for three times. After the last centrifugation, the pellets were re-suspended in 2 ml phosphate buffer (pH 7.2, 0.01 M containing 1% BSA and 0.05% sodium azide). Finally, NMBs-PAb was stored at 4 °C before being used (16).

### Sandwich ELISA

After several standardization trials, the following sandwich ELISA originally (17) was performed.

### Detection of Giardia antigens in stool by Dot-ELISA

Dot-ELISA was conducted as described earlier by (18) and modified by (19) after many standardization steps. Five μl of the IgG Ab containing 2 μg dotted on nitrocellulose membrane discs and allowed to be dried thoroughly. The discs were placed into flat bottom microplate wells. Non-specific binding sites were blocked by adding 100 μL of 2.5% FCS tris buffer solution containing 0.5% Tween 20 (TBST) to each well. Blocking solution was then aspirated off and antigen disks were washed by shaking (three times, 10 min each) with PBS with 0.05% Tween 20 (Riedel de Haen, AG, Seelze, Hanover, Germany) in TBS (vol/vol). Five microliters of prepared stool samples were added to each disk and incubated for 1 h at room temperature. The discs were washed again with PBST. The washing solution was removed and 2 μg/5 μL of horseradish peroxidase-labeled conjugate diluted in PBST was added to each well and incubated for 1 h at room temperature. The conjugate was removed and other washings were conducted as mentioned before. Ten mL of DAB substrate (diaminobenzidine tetrahydrochloride) (Sigma) was added to the nitrocellulose membrane and incubated for 2 min at room temperature. The development of a deep brown color dot on disks, when compared with negative serum control, was considered as evidence of positivity. Color development in control wells was completely absent or negligible.

### Detection of Giardia antigens in stool by NMB-dot ELISA

This method was performed, according to the original method of sandwich-dot ELISA (18) with some modification (19,20). The same as sandwich dot-ELISA but with the use of NMBs-PAb as a coating capture antibodies and NMBs-HRP-PAb as the conjugate detecting antibodies.

### Statistical analysis

All data were submitted to Microsoft Excel 2010 and analyzed using SPSS (Chicago, IL, USA). Results of nano-based Dot-ELISA were evaluated compared to the results of Dot-ELISA. Microscopic examination was taken as a gold standard and the diagnostic sensitivity, specificity; precision, positive predictive value and negative predictive value of the assay were calculated (21):

Sensitivity = TP × 100 / TP + FN
Specificity = TN × 100 / TN + FP
Precision = TN + TP × 100/ N
Positive predictive value = TP × 100/ TP + FP
Negative predictive value = TN × 100/ TN + FN

Where TP is true positive, TN is true negative, FP is false positive, FN is false negative and N is total sample

## Results

### Preparation and purification of G. lamblia antigen

**Fig. 1:**
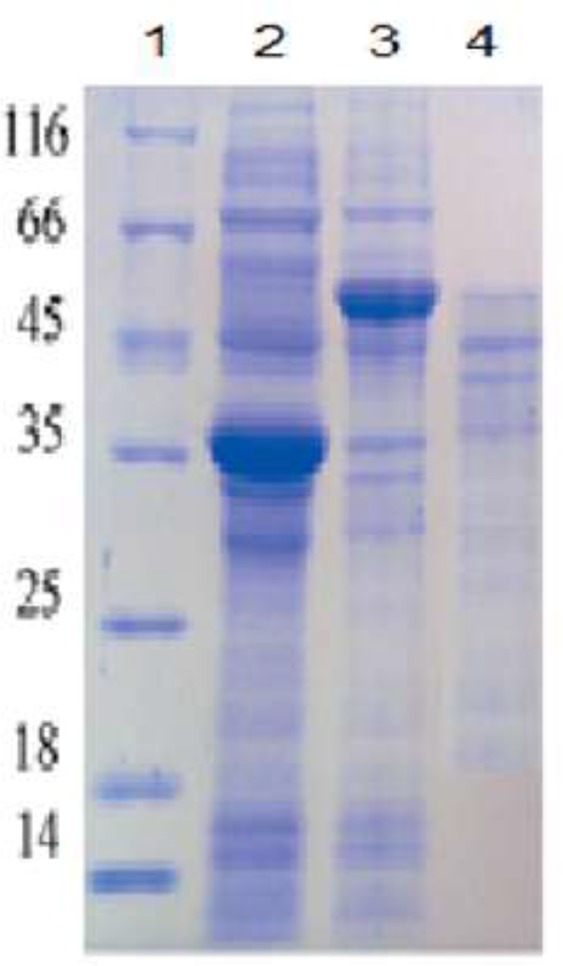
12% SDS-PAGE of *G. Lamblia* antigen under reduced conditions (Stained with Coomassie blue stain)
Standardcrude *Giardia* cystsPartially purified *Giardia* cystsPurified *Giardia* antigen

The SDS-PAGE analysis and Coomassie brilliant blue staining of *G. lamblia antigen* were shown in Fig. 1. The crude *G. lamblia* antigen containing several polypeptide bands ranged from 116 to 12 KDa. After first step of purification using Two-phase gradient technique many major bands, 55, 45, 37, 14 and 12 kDa were recognized. The purified *G. lamblia* antigen showed three major bands at 45, 42 and 37 kDa (Fig. 1).

### Reactivity and specificity of purified Giardia Antigen by Indirect ELISA

The antigenicity and specificity of the purified *Giardia* antigen were characterized by indirect ELISA. Stool samples from *G. lamblia* infected patients gave a strong reaction against purified *Giardia* antigen with mean OD reading equal to 0.977 and no cross-reactions were recorded with sample of patients infected with other parasites e.g., *E. histolytica* with mean OD reading equal to 0.11 and *cryptosporidium* with mean OD reading equal to 0.213.

### Standardization of Dot-sandwich ELISA for detection of Giardia Antigen

Determination of the optimum concentration of coating with anti- *Giardia* IgG polyclonal antibodies in dot-sandwich ELISA

The optimum concentration of coating anti- *Giardia* IgG polyclonal antibodies display in the highest OD reading at 492 nm against different concentrations of *Giardia* antigen (5, 10, 20, 40, 80 ug/ml) after the subtraction of background was found to be 20 μg/ml in sandwich ELISA the OD value =2.02.

### Determination of the optimum concentration of coating with anti- G. lamblia IgG pAb

**Fig. 2:**
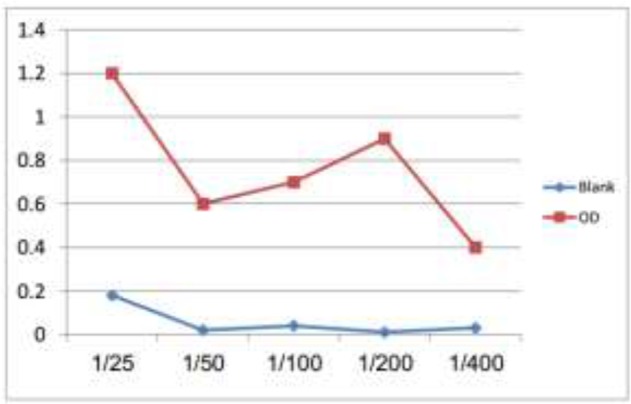
Determination of the optimum concentration of purified anti-*Giardia* IgG polyclonal antibodies as a conjugated layer in dot-sandwich ELISA

The optimum concentration of coating anti-*G. lamblia* IgG polyclonal antibody (evaluated by ELISA) displayed in the highest OD reading against different concentrations of *G. lamblia* antigen after the subtraction of background was found to be 1/25 (Fig. 2).

### Determination of the optimum concentration anti-G. lamblia conjugate

**Fig. 3:**
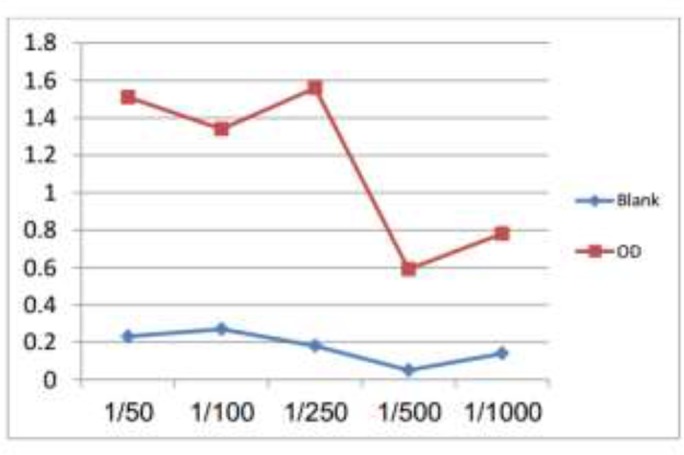
Determination of the optimum concentration of purified anti-*Giardia* IgG polyclonal antibodies as a conjugated layer in nano dot-sandwich ELISA

Anti-*G. lamblia* IgG pAb antibody was assessed against *Giardia* antigen in ELISA assay to determine the optimum working dilution of the conjugate. The results of titration, from which the 1/250 concentration of the conjugate gave the highest OD reading against Giardia antigen after subtraction of the background and was chosen as working dilution for subsequent assays (Fig. 3).

Dot ELISA for Detection of *G. lamblia* antigen in stool of patients: Six out of 32 *G. lamblia* infected samples from the (Group I) showed false negative results and the sensitivity of the assay was 81.3%. All the 20 negative controls were negative, while one out of twenty of other parasites groups gave false positive result, giving 97.5% specificity. The PPV and NPV were 86.3%, 96.7%, respectively (Tables 1, 2).

**Table 1:** Results of Dot-ELISA and NMB-Dot-ELISA for diagnosis of *Giardia lamblia*

***Variable Groups***	***Dot-ELISA***				***NMB-Dot-ELISA***			
	***Positive cases***		***Negative cases***		***Positive cases***		***Negative cases***	
	***Color range***	***No.***	***Color range***	***No.***	***Color range***	***No.***	***Color range***	***No.***
Healthy Control (n=20)	-	0	-	20	-	0	-	20
*Giardia* (n=32)		26		6	31	-		1
Light score	++	11	-	-	++	14	-	-
Moderate score	+++	7	-	-	+++	8	-	-
High score	++++	8	-	-	++++	9	-	-
Other parasites (n=20)		1		19		1		19
*E. histolytica (n=7)*	+	1	-	6	+	1	-	6
*E. Coli (n=7)*	-	0	-	7	-	0	-	7
*Toxoplasma (n=6)*	-	0	-	6	-	0	-	6

**Table 2:** Diagnostic accuracy of Dot-ELISA and NMB-Dot-ELISA in diagnosis of *G. lamblia* infection

***Variable***	***Dot-ELISA***	***NMB-Dot-ELISA***
Sensitivity	81.3	96.9
Specificity	97.5	97.5
Precision	90.3	97.2
Positive predictive value	96.3	96.9
Negative predictive value	86.7	97.5

NMB-dot ELISA for detection of *G. lamblia* antigen in stool of infected patients: Only one out of 32 *G. lamblia* infected samples (Group I) showed false negative results and the sensitivity of the assay was 96.7%. All the 20 negative controls were negative color range, while one out of twenty of the other parasites group gave false positive result giving 97.5% specificity. The PPV and NPV were 96.9% and 97.5%, respectively (Tables 1, 2).

## Discussion

*Giardia lamblia* is included in the “Neglected Disease Initiative” of WHO because of its impact on socioeconomic development especially in developing countries (22). Diagnosis of giardiasis is usually based on microscopic examination of stool samples by visualizing the organism (23). Single stool specimen examination has a low sensitivity and may miss up to 50% of *Giardia* infections (24) particularly if there is a low parasite density, intermittent parasite excretion. Although new approaches to the diagnosis of giardiasis have been established, the majority of clinical laboratories especially commercial ones still depend on routine microscopy for demonstration of *Giardia lamblia* in feces and small intestinal specimens (25). Being presents a highly variable excretion pattern, *Giardia lamblia* parasite can be easily misdiagnosed sure positive cases. In addition, proper microscopic examination of multiple stool samples necessitates generous examination time per each sample, qualified personnel for getting a reliable & quality control results. Therefore, the development of specific, sensitive, and reliable techniques to demonstrate the presence of *G. lamblia* coproantigen is an important step towards improving its diagnosis.

Dot enzyme-linked immunosorbent assay (Dot-ELISA) is a versatile solid-phase immunoassay for antibody or antigen detection. It uses minute amounts of reagent dotted onto solid surfaces which avidly bind proteins. After incubation with antigen-specific antibody and enzyme-conjugated anti-antibody, adding of chromogenic substrate causes formation of a colored dot visually read (5,6). Herein, we developed a new NMB dot ELISA and compared to traditional dot ELISA for diagnosis of *G. lamblia*. Our work has demonstrated that the novel nano-immunodiagnostic assay conferred a higher sensitivity (96.9%) in detecting *G. lambilia* in stool samples compared with dot ELISA (81.3%). Moreover, the NMB dot ELISA gave higher PPV and NPV (96.9% and 95.5% respectively) than the dot ELISA (96.3%, and 86.7%, respectively). Sensitivity and specificity of the traditional sandwich ELISA test were reported as 76.4% and 100% respectively in the detection of *G. lamblia* antigen in stool specimens which is in accordance with our results (26). Traditional ELISA showed the sensitivity, the specificity, negative and positive predictive values of 100%, 96.15%, 100%, and 80%, respectively when microscopy was used as the reference standard, the higher sensitivity of their assay may be attributed to type of antigen used or fresh versus frozen samples (27). Many authors reported different sensitivity & specificity of ELISA (28–30). A considerable discrepancy in performance of 3 commercially available ELISA kits in detection of Giardia antigen was observed according to the preservative used in sampling which denotes that antigen level in stool samples could be affected according to preparation procedure and may explain the difference between this study results and former ones as regard traditional sandwich ELISA (31).

Magnetic nanoparticles have been reported to have promising applications in medical diagnostics and therapy (32) as well as immunoassay based diagnostics (33). In the immunoassay diagnostics, the functionalized magnetic particle bound to a biomolecule recognition unit is used as a label instead of enzymes or fluorescent material to capture the target analyte (34). Herein, we developed a new NMB dot ELISA and compared to traditional dot ELISA for diagnosis of *G. lamblia*. Our work has demonstrated that the novel nano-immunodiagnostic assay conferred a higher sensitivity (96.9%) in detecting *G. lambilia* in stool samples compared with dot ELISA (81.3%). Moreover, the NMB dot ELISA gave higher PPV and NPV (96.9% and 95.5% respectively) than the dot ELISA (96.3%, and 86.7%, respectively).

Nanogold beads sandwich ELISA was used. Nanogold beads-sandwich ELISA sensitivity was 95.8% compared with the traditional sandwich ELISA (93%) (8). Moreover, nano-sandwich ELISA gave higher specificity, PPV and NPV (95%, 97.2% and 92.6%, respectively) than sandwich ELISA (92.5%, 95.7% and 88%, respectively). Their results using nanogold beads had lower sensitivity and PPV than our results obtained by using nanomagnetic beads. The better diagnostic parameters of the nano-magnetic beads are due to their high binding capacity (as a solid phase) and rapid reaction of solutions thus, they enhance the antigen detection in the immunoassay.

## Conclusion

Diagnosis of *G. lamblia* by NMB-Dot-ELISA technique is sensitive, specific, rapid, and easy to perform. In addition, the Nano-based-Dot-ELISA may be configured to detect parasite antigen or antibodies in either microtiter plates or dipsticks.
